# Enhanced Arsenic (III and V) Removal in Anoxic Environments by Hierarchically Structured Citrate/FeCO_3_ Nanocomposites

**DOI:** 10.3390/nano10091773

**Published:** 2020-09-08

**Authors:** Seon Yong Lee, YoungJae Kim, Bongsu Chang, Young Jae Lee

**Affiliations:** 1 Department of Earth and Environmental Sciences, Korea University, 145 Anam-ro, Seongbuk-gu, Seoul 02841, Korea; reddevels86@korea.ac.kr (S.Y.L.); bschang@korea.ac.kr (B.C.); 2 Chemical Sciences and Engineering Division, Argonne National Laboratory, 9700 South Cass Avenue, Lemont, IL 60439, USA; youngjkm@anl.gov

**Keywords:** siderite, citrate, nanocomposite, arsenic removal, anoxic environment

## Abstract

Novel citrate/FeCO_3_ nanocomposites (CF-NCs) were synthesized for effective arsenic (III and V) sorption with constant addition of Fe^2+^ into HCO_3_^−^ solution in the presence of citrate. This paper is the first report on the formation of CF-NCs, and in this study we investigate the mechanisms of arsenic uptake by the sorbent under anoxic conditions through various solid- and liquid-phase spectroscopic methods, including X-ray absorption spectroscopy. In CF-NCs, citrate was found to be incorporated into the structure of siderite (up to 17.94%) through (Fe^2+^citrate)^−^ complexes. The crystal morphology of rhombohedral siderite was changed into hierarchically nanostructured spherical aggregates composed of several sheet-like crystals, which improved the surface reactivity in the presence of sufficient citrate. Compared to pure siderite (15.2%), enhanced removal of As(III) in the range of 19.3% to 88.2% was observed, depending on the amount of incorporated citrate. The maximum sorption capacities of CF-NCs for As(III) and As(V) were 188.97 and 290.22 mg/g, respectively, which are much higher than those of previously reported siderite-based adsorbents. It was found that arsenic (III and V) sorption on CF-NCs occurred via bidentate corner-sharing surface complexation, predominantly without changes in the arsenic oxidation states. These results suggest that arsenic (III and V) can be attenuated by siderite in anoxic environments, and this attenuation can be even more effective when siderite is modified by incorporation of organic compounds such as citrate.

## 1. Introduction

Arsenic (As) is a ubiquitous element that occurs naturally in the Earth’s crust as a constituent of over 245 minerals generated through geological processes [1,2,3]. However, anthropogenic activities such as mining, the use of fossil fuels, glass manufacturing, and the application of arsenic-containing pesticides have resulted in arsenic contamination [2,3]. Furthermore, it has been established that long-term exposure to arsenic in drinking water can cause chronic health problems such as skin, lung, liver, prostate, bladder, and kidney cancers [4,5,6]. To protect public health, many countries, including the United States and the European Union, have adopted a new concentration limit of 10 μg/L of As in drinking water [7,8]. However, arsenic contamination in groundwater remains a serious problem in many Asian countries such as Bangladesh, Vietnam, India, and China [9,10,11,12,13].

While As has four oxidation states (i.e., V, III, 0, and −III) depending on environmental parameters such as pH and redox potentials, two arsenic species, inorganic arsenite (As(III)) and arsenate (As(V)), are dominant in natural water [14]. As(III) species (e.g., H_3_AsO_3_^0^) are known to be approximately 60 times more toxic than As(V) species (e.g., HAsO_4_^2−^ and H_2_AsO_4_^−^) [15,16], and unfortunately they are also the dominant form in most arsenic-contaminated groundwater resources owing to the limited supply of oxygen in subsurface environments [3,17,18,19,20]. Compared to As(V), the attenuation of neutrally charged As(III) species has been challenging owing to their weak electrostatic attraction towards sorbents in a neutral pH solution [2,21]. Research on the enhanced sorption of As(III) is therefore important to achieve the effective attenuation of arsenic in groundwater and other anoxic environments.

Recently, siderite (FeCO_3_) has received considerable attention as a promising arsenic sorbent which is both cost-effective and environmentally harmless. In particular, it features high surface reactivity [22,23,24,25,26,27,28,29] and remains stable in anoxic-reducing systems where ferrous iron, carbonate, and organics are abundant [30,31,32]. For these reasons, it has been deemed a suitable material for attenuating As(III) in subsurface environments. However, according to earlier studies on As(III) sorption on siderite in anoxic conditions, the sorption performance of siderite for As(III) is very low compared to that for As(V) [24,28]. Therefore, it is necessary to improve the sorption capacity of siderite for practical use as an effective sorbent in anoxic environments.

Siderite commonly occurs in groundwater-fed wetland sediments that are associated with organic-rich water. In fact, such environments can provide suitable conditions for siderite formation through the reduction of ferric iron and a sufficient supply of carbonate from organic matter decomposition [32,33]. The interaction of siderite with organic ligands may therefore play an important role in controlling crystal growth and the physicochemical properties of carbonate minerals (e.g., crystal morphology, its solubility, and possibly the sorption ability for contaminants) [34,35]. Citric acid (HOC(COOH)(CH_2_COOH)_2_) is a common organic ligand in natural water and is also considered as a model compound for humic substances, having three carboxylic groups and one hydroxyl group [36]. While citrate inhibits the crystallization of carbonate minerals owing to the adsorption of citrate on the mineral surface [37,38], it is known to be incorporated into the structure of carbonates [38,39]. It is also known that this surface modification by organic functional groups (such as carboxylic ligands) can improve the reactivity of minerals [40] and thus be employed for developing a novel As(III) detector with high sensitivity [41].

This study is the first in which a siderite-based highly reactive sorbent was synthesized in the presence of citrate and its efficiency in removing As was evaluated. Citrate incorporation into siderite and arsenic sorption mechanisms were systematically investigated based on microscopic and spectroscopic measurements, including synchrotron X-ray techniques. The results of this study provide comprehensive mechanistic insights into the formation of citrate/FeCO_3_ nanocomposites as well as the enhanced arsenic sorption on the new material in anoxic environments.

## 2. Materials and Methods

### 2.1. Materials

The following analytical grade reagents were purchased from Sigma-Aldrich: FeCl_2_·4H_2_O, NaHCO_3_, and citric acid for the synthesis of citrate/FeCO_3_ nanocomposites (hereinafter referred to as “CF-NCs”); Na_2_HAsO_4_·7H_2_O and NaAsO_2_ for batch sorption experiments; and 37% HCl, 70% HNO_3_, and 50% NaOH solutions for pH adjustment. Dissolved oxygen (DO)-free water ([DO] < 0.00 ppm) was used in all the synthetic and sorption processes. The DO-free water was prepared as follows. First, deionized water ([DO] = 6–7 ppm) with a resistance of 18.2 mΩ (Milli-Q Plus, Merck KGaA, Darmstadt, Germany) was boiled in an electric kettle, placed into a 500 mL sealing container, and cooled with 99.999% N_2_ purging for 20 min. Then, the container was placed in an anaerobic chamber (Vinyl Type B, Coy Laboratory Products Inc., Grass Lake, MI, USA) and opened for additional sparging with a H_2_/N_2_ mixture (6% H_2_) using a micro-bubbler for 20 min. Inside the anaerobic chamber, 1 M HCl and NaOH solutions for pH adjustment and As(III) and As(V) stock solutions of 1000 mg/L for the designed sorption experiments were prepared in advance, whereas Fe^2+^ and HCO_3_^−^ solutions were prepared immediately before the synthesis experiment.

### 2.2. Synthesis of the Citrate/FeCO_3_ Nanocomposites

All synthetic experiments were performed at 25 ± 0.1 °C, 40–60% relative humidity, and 1 atm with 5–600 ppm of CO_2_ (measured by IAQ-CALCTM 7525, TSI Incorporated, Shoreview, MN, USA) inside an anaerobic chamber. The chamber atmosphere, periodically filled with an H_2_/N_2_ mixture (6% H_2_), was continuously circulated through palladium catalyst fans to reduce the trace O_2_ and H_2_O generated by reaction (1), similar to the method reported in [42]:

2H_2_ + O_2_ → 2H_2_O (1)

The anaerobic chamber was also equipped with a gas analyzer to monitor O_2_ and H_2_, and their levels were kept at O_2(g)_ ≤ 1 ppm and H_2(g)_ > 2.0% throughout each experiment. The atmospheric and solution temperatures were maintained using two palladium catalysts fan boxes (Stak-Pak) with a temperature controller (±1 °C) and a hotplate magnetic stirrer with a temperature sensor (± 0.1 °C) (C-MAG HS7/ETS-D5, IKA, Staufen, Germany). Important solution chemistry parameters of the desired solution, such as pH, oxidation reduction potential (ORP), and DO concentration, were measured using a pH meter (Orion Versastar, Thermo Scientific, Waltham, MA, USA) equipped with pH, ORP, and DO electrodes (Orion 8156 BNUMD Ross Ultra pH/ATC Triode, Orion 9179BNMD, and Orion 087003).

Citrate/FeCO_3_ nanocomposites (CF-NCs) were synthesized with different citric acid concentrations (0.5, 1.0, 2.0, 2.5, 3.0, 4.0, and 5.0 mM) using a drop-wise constant addition method with a multi-syringe pump. A freshly prepared solution of 100 mM Fe^2+^ (240 mL with citrate; pH 2.4) was added to a 100 mM HCO_3_^−^ solution (240 mL with citrate; pH 7.0) for 4 h with continuous stirring, and the mixture was then aged for 24 h with stirring. Immediately after aging, the precipitate in the solution was separated using a 0.2 μm membrane filter and thoroughly rinsed with DO-free water to remove any residual ions. The filtered solid was placed and dried on a petri dish at 50 °C for 1 day and then stored in a silicagel-containing vacuum desiccator for further characterization and sorption experiments. A schematic of the CF-NCs synthesis procedure is shown in Figure S1.

### 2.3. Batch Sorption Experiments

The prepared arsenic (III, V) stock solutions were used for sorption experiments after dilution to the desired concentrations using DO-free water with pH 7.0. A synthesized solid sample of 0.04 g was put into 40 mL (particle loading = 1 g/L) of 50 mg/L arsenic solution and reacted in an orbit shaking incubator (LSI-3016R, Daihan LabTech Co., Namyangju-si, Korea) at 25 ± 0.1 °C and 200 rpm. The first As(III) sorption experiment was performed using CF-NCs synthesized at different citrate concentrations (0–5 mM) to confirm the effects of citrate on As(III) removal and determine the appropriate citrate concentration. Then, arsenic (III, V) sorption kinetics were evaluated for the CF-NCs, which exhibited the highest removal rate of As(III), in 50 mg/L arsenic solution with a particle loading of 1 g/L at 25 °C for 48 h. Sorption isotherms were obtained with varying arsenic concentrations from 1 to 500 mg/L at 25 °C for 48 h. When the reaction was completed, an aliquot was extracted from each sample and filtered using a syringe filter (0.2 μm), followed by acidification using 100 μL of 70% HNO_3_ for further analysis. The removal rate (*R*, %) and sorption capacity (*q_e_*, mg/g) at equilibrium were determined using the residual arsenic concentration and the following equations:

(2) $$R\;(\%)\;=\;\frac{\left(C_{i}\;-\;C_{e}\right)}{C_{i}}\times 100$$

(3) $$q_{e}=\frac{V}{M}(C_{i}-C_{e})$$

where *C_i_* and *C_e_* are the initial and equilibrium concentrations of arsenic (mg/L), respectively, *q_e_* is the weight of arsenic sorbed per unit weight of the sorbent (mg/g), *V* is the volume of the solution (L), and *M* is the mass of the sorbent (g).

The pseudo-first-order and pseudo-second-order kinetic sorption models can be represented as follows:

(4) $$\mathrm{Pseudo}\text{-}\mathrm{first}\text{-}\mathrm{order}\;q_{t}=q_{e}(1-e^{-k_{1}t})$$

(5) $$\mathrm{Pseudo}\text{-}\mathrm{second}\text{-}\mathrm{order}\;q_{t}=\frac{k_{2}q_{e}^{2}t}{1+k_{2}q_{e}t}$$

where *q_t_* and *q_e_* are the sorbed amounts of arsenic (mg/g) at a given contact time (*t*, h) and equilibrium, respectively, and *k*_1_ (1/h) and *k*_2_ (g/mg h) are the rate constants of pseudo-first-order and pseudo-second-order, respectively. The coefficient of determination (*R*^2^) and an error function (root mean square error, RMSE) were applied as criteria to quantitatively evaluate the best fit. They are expressed as follows:

(6) $$\mathrm{Coefficient}\;\mathrm{of}\;\mathrm{determination}\;R^{2}=\frac{1-\sum_{n=1}^{n}{\left(q_{e,\exp,n}-q_{e,\mathrm{cal},n}\right)}^{2}}{\sum_{n=1}^{n}{\left(q_{e,\exp,n}-\bar{q_{e,\mathrm{cal},n}}\right)}^{2}}$$

(7) $$\mathrm{Root}\;\mathrm{mean}\;\mathrm{square}\;\mathrm{error}\;(\mathrm{RMSE})\;\mathrm{RMSE}=\sqrt{\frac{1}{n-1}\sum_{n=1}^{n}{\left(q_{e,\exp,n}-q_{e,\mathrm{cal},n}\right)}^{2}}$$

where *q_e,exp_* and *q_e,cal_* are the experimental and calculated adsorption capacities, and *n* is the number of observations.

The Langmuir and Freundlich sorption isotherm models can be expressed as follows:

(8) $$\mathrm{Langmuir}\;q_{e}=\frac{q_{m}k_{L}C_{e}}{1+k_{L}C_{e}}$$

(9) $$\mathrm{Freundlich}\;q_{e}=k_{F}C_{e}^{1/n}$$

where *C_e_* is the equilibrium concentration of arsenic (mg/L), *q_m_* is the theoretical maximum adsorption capacity (mg/g), *k_L_* and *k_F_* are the Langmuir (L/mg) and Freundlich (mg/g (L/mg)^1/n^) constants, respectively, and 1/*n* is the adsorption intensity.

### 2.4. Characterization

#### 2.4.1. Solid- and Liquid-Phase Analyses

To prevent possible oxidation, all samples were thoroughly sealed in an anaerobic chamber, transported to the analytical instruments using an anaerobic jar with an anaerobic gas pack, and opened just before the analysis. After the batch sorption experiments, the residual concentrations of Fe and As were analyzed by inductively coupled plasma spectrometry (ICP-OES, ICAP 7200, Thermo Scientific, Waltham, MA, USA). Citric acid concentrations in the solid and solution were measured using high-performance liquid chromatography (HPLC) (1200 series, Agilent Technologies, Waldbronn, Germany) after acidification by 1 M HCl. X-ray diffraction (XRD) patterns for the as-synthesized samples were measured using a diffractometer (SmartLab, Rigaku, Tokyo, Japan) with CuKα radiation at a scan rate of 1.0°/min in the 2θ range of 15–65°. Scanning electron microscopy (SEM) micro-images of the samples were obtained using a scanning electron microscope (Quanta 250FEG, FEI, Hillsboro, OR, USA) operated at an accelerating voltage of 10.0–15.0 kV. The localized elemental compositions were simultaneously analyzed by energy dispersive X-ray spectroscopy (EDS). Fourier transform-infrared (FT-IR) spectra were obtained in transmission mode with a range of 4000–400 cm^−1^ on a Cary 630 ATR-FTIR spectrometer (Agilent Technologies, Inc., Santa Clara, CA USA). Thermogravimetric and differential scanning calorimetry (TG-DSC) analyses were performed using a thermal gravimetric analyzer (SDT Q600, TA Instruments, New Castle, DE, USA) at a heating rate of 5 °C/min. X-ray photoelectron spectroscopy (XPS) spectra of each sample were obtained using X-ray photoelectron spectroscope (X-TOOL, ULVAC-PHI, Chigasaki, Japan) and calibrated using the C1s spectral component (C–C) with a binding energy of 284.8 eV. In addition, speciation modeling of the synthetic system was performed using PHREEQC software (version 2.18, U.S. Geological Survey, Reston, VA, USA) to explain the property of citrate incorporation into siderite and support the suggested formation mechanisms of citrate/FeCO_3_ nanocomposites.

#### 2.4.2. X-ray Absorption Spectroscopy (XAS)

For the sample preparation for X-ray absorption near edge structure (XANES) and extended X-ray absorption fine structure (EXAFS) analyses, the arsenic-sorbed solid samples were separated using a 0.2 μm membrane filter after the sorption process reached the equilibrium. Then, they were immediately loaded into acrylic sample holders and sealed with Kapton tape to prevent contact with air and drying. Arsenic reference compounds (Na_2_HAsO_4_·7H_2_O and NaAsO_2_) were mixed with boron nitride to obtain a proper EXAFS signal and then sealed using Kapton tape. All samples were stored in anaerobic jars with an anaerobic pack and transferred to the beamline 8C-Nano XAFS (Pohang Accelerator Laboratory, Pohang-si, Korea). EXAFS spectra were collected at the arsenic K-edge (11.867 keV) using Si(111) crystal monochromators with 30% detuning for harmonic rejection. The monochromators were calibrated by assigning the indicated energies to the first peak of the derivative of the edge spectrum. Energy calibration was performed with Au metal foil at the L_3_ edge (11.919 keV). EXAFS data were obtained in transmission mode for the arsenic reference compounds and in fluorescence mode for the arsenic sorption samples using a partially implanted planar silicon detector and a multi-element Ge solid-state detector. Data processing and multi-parameter shell fitting were performed using WinXAS and Ifeffit software [43,44]. The EXAFS oscillation function was extracted in k-space (*k* = 2.7–12.0 Å^−1^), weighted by *k*^3^ for *k*^3^χ(*k*) functions, and then Fourier-transformed in a selected data range to generate radial structural functions (RSFs) to determine the geometrical parameters of the sorbed As atoms. Crystal information files of Na_2_HAsO_4_·7H_2_O and NaAsO_2_ were obtained from the American Mineralogist Crystal Structure Database (AMCSD) and used to calculate the theoretical models to be fitted to the individual RSFs from experimental data.

## 3. Results and Discussion

### 3.1. Characterization of Citrate/Siderite Nanocomposites

**Figure 1:**
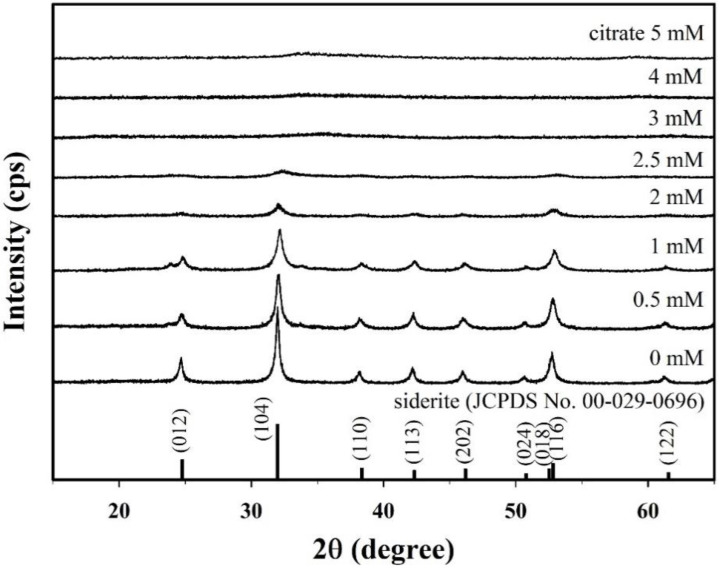
X-ray diffraction (XRD) patterns for the citrate/FeCO_3_ nanocomposites (CF-NCs) synthesized in the presence of different citrate concentrations (0–5 mM).

Figure 1 presents the X-ray diffraction (XRD) patterns of samples synthesized with different citrate concentrations (0–5 mM). The XRD peaks for the sample without citrate at 24.8°, 32.0°, 38.4°, 42.3°, 46.2°, 50.8°, 52.5°, 52.8°, and 61.5° correspond to the (012), (104), (110), (113), (202), (024), (018), (116), and (122) crystal planes of typical siderite (JCPDS No. 00-026-0696), respectively, which indicates that siderite was successfully formed without secondary phase under this synthetic condition. The saturation index (SI) of the synthetic solution with respect to siderite decreased slightly from 3.17 to 3.14 as the concentration of citrate increased. However, the solution remained supersaturated (Figure S2D), indicating that siderite can be stably formed. This is because the concentration of Fe^2+^ was significantly higher than that of citrate (Figure S2B). However, the intensities of the XRD peaks decreased gradually with the increasing citrate concentration, and all the peaks disappeared in the samples prepared at a citrate concentration of 3 mM or higher. This means that the crystallinity of the synthesized samples and their structural properties are greatly changed over a wide concentration range (0 to 5 mM) of citrate.

**Figure 2:**
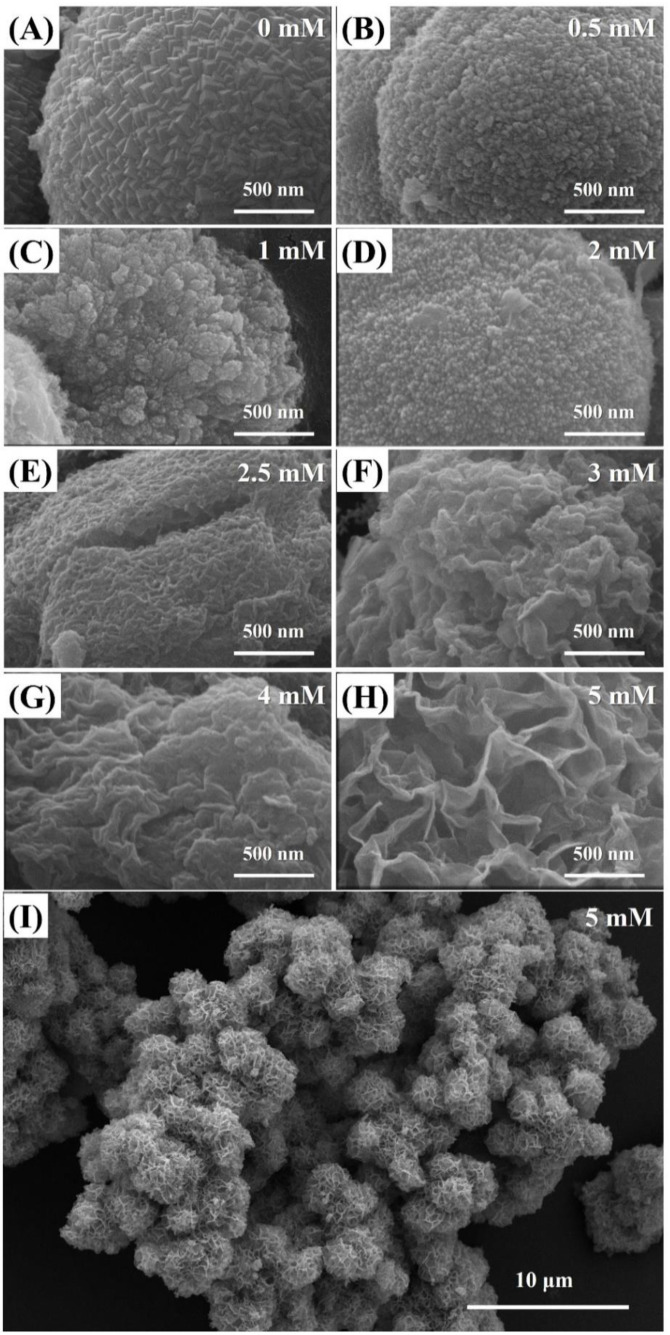
(**A**–**H**) Scanning electron microscopy (SEM) images for the CF-NCs synthesized in the presence of different citrate concentrations (0–5 mM); (**I**) is the low-magnification image of (**H**).

Scanning electron microscopy (SEM) images, presented in Figure 2, reveal that the morphological features of CF-NCs vary depending on the concentration of citrate. In a sample synthesized without citrate (Figure 2A), siderite appeared as spherical aggregates (2–4 μm in diameter) composed of nano-sized rhombohedral crystals. This is consistent with a previous report on micro-sized spheres of synthetic siderite [45]. The aggregates of siderite decreased in size and their characteristic rhombohedral shape vanished as the concentration of citrate increased from 0.5 mM to 2 mM (Figure 2B–D). The aggregated crystals were no longer spherical, and the individual crystal started to exhibit a crumpled texture at the surface with 2.5 mM of citrate (Figure 2E). The crumpled surface gradually developed to a prominent sheet-like structure as the citrate concentration increased further (Figure 2F,G) and finally showed a 3D structure composed of several sheet-like crystals with 5 mM of citrate (Figure 2H). These spherical particles form hierarchically nanostructured aggregates of a few tens of micrometers in size, which are known to be favorable for adsorption and/or catalytic reactions because of their improved pore structure (Figure 2I) [46,47].

**Figure 3:**
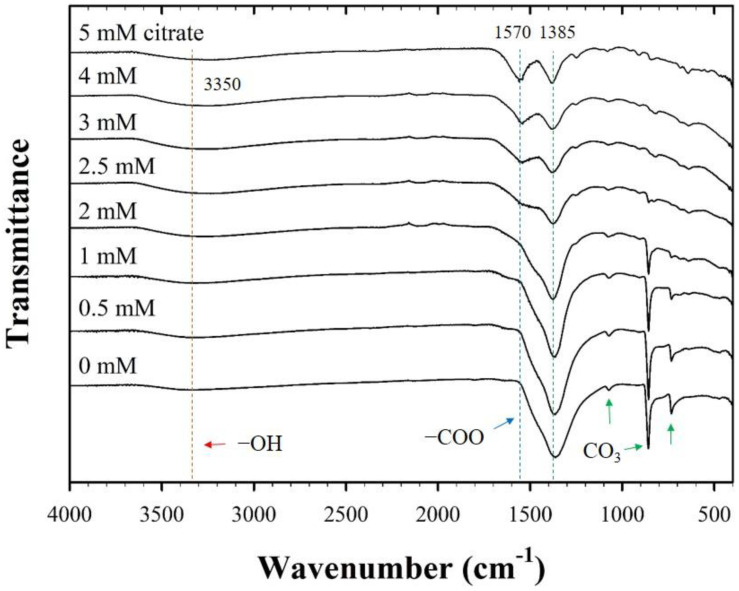
Fourier transform-infrared (FT-IR) spectra for the CF-NCs synthesized in the presence of different citrate concentrations (0–5 mM).

The FT-IR spectra also exhibited significant changes in the surface functionality of CF-NCs owing to the presence of citrate (Figure 3). For pure siderite, FT-IR peaks were attributed to the stretching and bending vibration bands of CO_3_ at 1385, 1070, 862, and 737 cm^−1^, which are in agreement with those reported for siderite in previous studies [48,49,50,51]. In samples with citrate, two additional peaks found at ~3350 cm^−1^ and 1570 cm^−1^ are assigned to the symmetric vibration of the –OH group from water and citrate and the asymmetric stretching vibration of the carboxyl/carbonyl group (–COO) of citrate, respectively [52]. The intensities of the former peaks belonging to siderite decreased, and those of the latter peaks related to citrate increased, as the concentration of citrate increased. According to previous reports, Fe(II)-citrate_(s)_ has a water molecule in the structure (FeC_6_H_6_O_7_·H_2_O) [53,54], which may relate to the structural distortion of the siderite. This indicates that the strong peak observed at ~3350 cm^−1^ in the FT-IR spectra of CF-NCs is associated with the presence of the Fe-citrate complex in the samples. Furthermore, the gradual increase in the new citrate peak and the decrease in the CO_3_ peak of the samples with increasing citrate concentration may indicate citrate incorporation into siderite, which is facilitated if the concentration of citrate is high.

**Figure 4:**
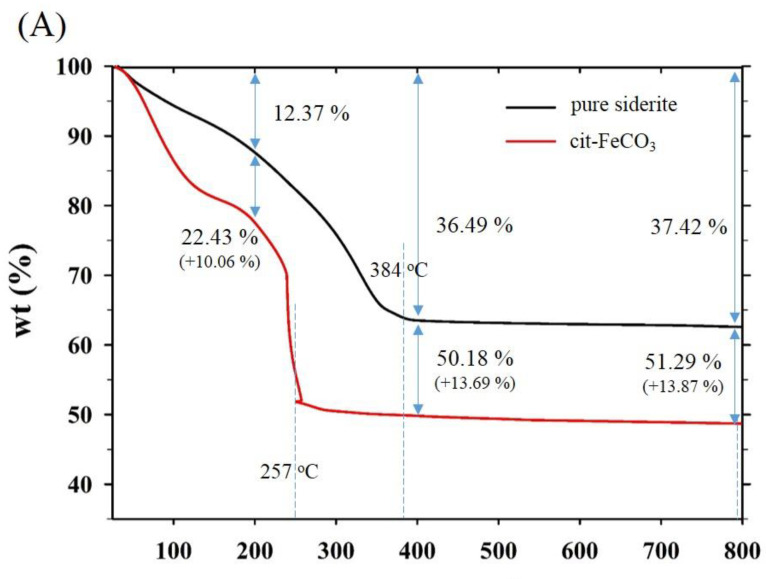

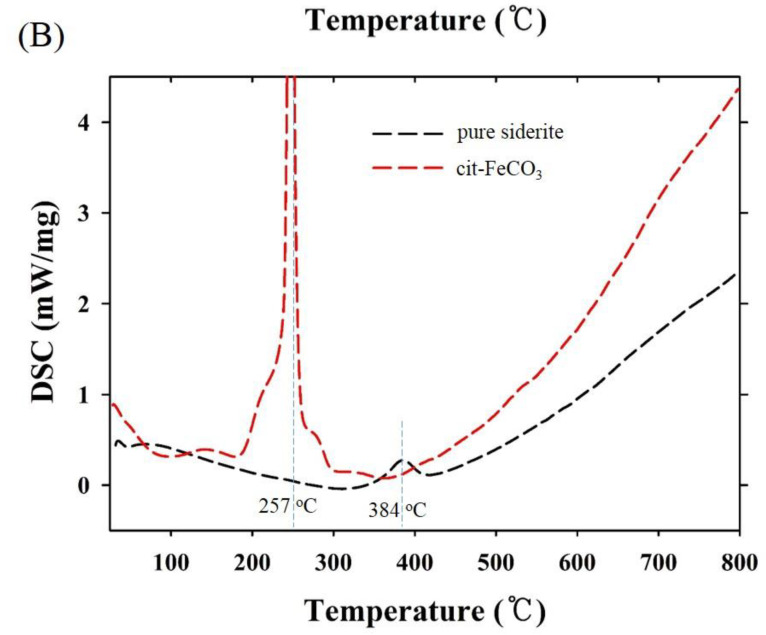
(**A**) Thermogravimetric (TG) and (**B**) differential scanning calorimetry (DSC) profiles of the two CF-NCs synthesized at 0 mM and 5 mM citrate concentration, respectively.

Thermogravimetric and differential scanning calorimetry (TG-DSC) profiles of the two samples synthesized at 0 mM (i.e., pure siderite) and 5 mM of citrate (CF-NCs) are presented in Figure 4. During the TG analysis, pure siderite exhibited a gradual weight loss of 36.49% up to 400 °C owing to dehydration and decomposition of siderite into iron oxides and CO_2_ [55,56,57], whereas CF-NCs exhibited a greater weight loss (13.69%) than pure siderite. Considering their DSC profiles, an endothermic peak at 384 °C in the profile of pure siderite resulted from the typical transformation of siderite to other iron oxides (e.g., magnetite), whereas the strong and sharp peak at 257 °C (in the range 180–300 °C) in the DSC profile of CF-NCs could be attributed to the decomposition of citrate. These results indicate that a significant amount of citrate is present in the CF-NCs.

High-performance liquid chromatography (HPLC) results for samples synthesized at different citrate concentrations (0–5 mM) are presented in Table 1. Citrate in the samples increased from 2.28 wt.% to 17.94 wt.% as the initial citrate concentration increased from 0.5 mM to 5 mM. The citrate content steeply increased over a range of 2–3 mM of citrate.

### 3.2. Formation Mechanisms of Citrate/FeCO_3_ Nanocomposites

In this section, based on the XRD, FT-IR, TG-DSC, and SEM results, the formation mechanism(s) of citrate/FeCO_3_ composites (CF-NCs) as a function of citrate concentration is discussed in detail. When the CF-NCs were synthesized in the presence of higher citrate in solution, the crystallinity of siderite was significantly decreased, and the CO_3_ peak in the FT-IR spectrum gradually decreased, followed by an increase in the citrate peak. In addition, most of the citrate species exist as (Fe^2+^citrate)^−^ in solution (Figure S2C). According to Phillips et al. (2005) [35], citrate can be incorporated into the calcite (CaCO_3_) structure up to 1 wt.%, and the incorporation of a metal chelate complex like (Fe^2+^citrate)^−^ in the structure might be possible. This means that disorder in the lattice structure of siderite can be caused by the incorporation of the (Fe^2+^citrate)^−^. Based on these results, it can be concluded that citrate is not only adsorbed on the surface of siderite with the (Fe^2+^citrate)^−^ form but also incorporated into the siderite structure during siderite formation. The adsorption or incorporation of (Fe^2+^citrate)^−^ can hinder the growth of siderite crystals, which may be due to surface poisoning or/and structural distortion [37]. When the citrate concentration is increased, these effects become stronger, causing the formation of smaller siderite crystals, which are covered by many (Fe^2+^citrate)^−^ complexes. This may imply the possibility of precipitating the Fe(II)-citrate_(s)_ without forming siderite (FeCO_3_) crystal. However, considering the very high water solubility of the Fe(II)-citrate_(s)_ (5.03 g/L) [58,59], it is very difficult to cause the precipitation of the Fe(II)-citrate_(s)_ alone. Therefore, it is considered that the CF-NCs prepared at 5 mM of citrate were formed through the aggregation of several amorphous FeCO_3_ nanoparticles incorporated by sufficient (Fe^2+^citrate)^−^ complexes.

TG-DSC and HPLC results provided quantitative information on the citrate in the CF-NCs and also proved that a significant amount of citrate (up to 17.94 wt.%) can be incorporated into siderite (see Figure 4 and Table 1). Furthermore, it is notable that a sharp increase in the citrate content of CF-NCs occurred in a narrow concentration range (2–3 mM of citrate), as determined by HPLC (Table 1). This significant change was consistent with changes in the crystallinity, morphology, and surface functionality of the CF-NCs synthesized at the same loading of 2–3 mM of citrate. These observations suggest that a concentration of 2–3 mM of citrate is a critical condition controlling the physicochemical properties of CF-NCs.

**Figure 5:**
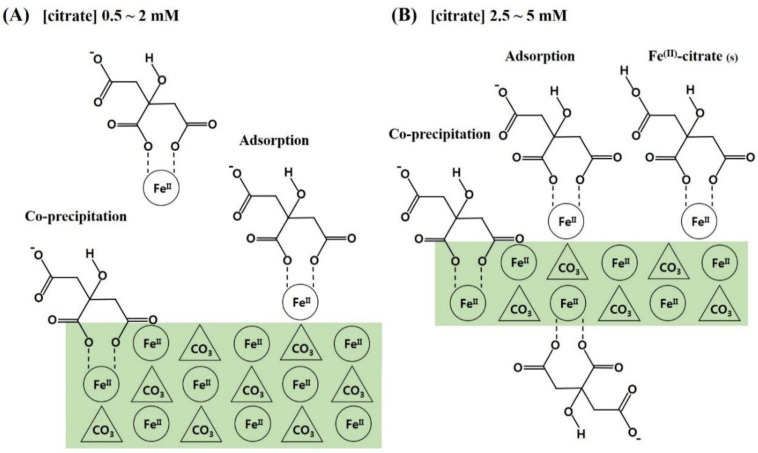
Schematic illustrations for the formation of citrate/FeCO_3_ nanocomposites (CF-NCs). (**A**) Adsorption and co-precipitation of (Fe^2+^citrate)^−^ complexes at the surface of larger siderite crystal, (**B**) Adsorption and co-precipitation of (Fe^2+^citrate)^−^ complexes at the surface of smaller siderite crystallite with surface precipitation of some Fe(II)-citrate_(s)_ (modified from Lee and Reeder, 2006) [38].

Based on these results, the following conclusions were drawn and are schematically presented in Figure 5: (1) Citrate is favorably incorporated into siderite through the form of (Fe^2+^citrate)^−^ complex, and citrate incorporation is greatly influenced by the loading concentration of citrate in the synthetic solution; (2) the incorporation of citrate occurs through adsorption of the (Fe^2+^citrate)^−^ complex and co-precipitation, which results in retardation of crystal growth for siderite with significant changes in surface functionalities and crystal morphologies, providing siderite with a hierarchical nanostructure and sufficient functional groups; (3) these effects increase critically in the 2–3 mM concentration range of citrate and are maximal at 5 mM of citrate, which improves the physicochemical properties of the sorbent. Therefore, these findings indicate that the synthesis of novel nanocomposites by citrate incorporation into siderite is an important strategy for effective As(III) removal under anoxic subsurface environments.

### 3.3. Batch Sorption Results

#### 3.3.1. Effects of Citrate Incorporated into CF-NCs on As(III) Sorption

**Figure 6:**
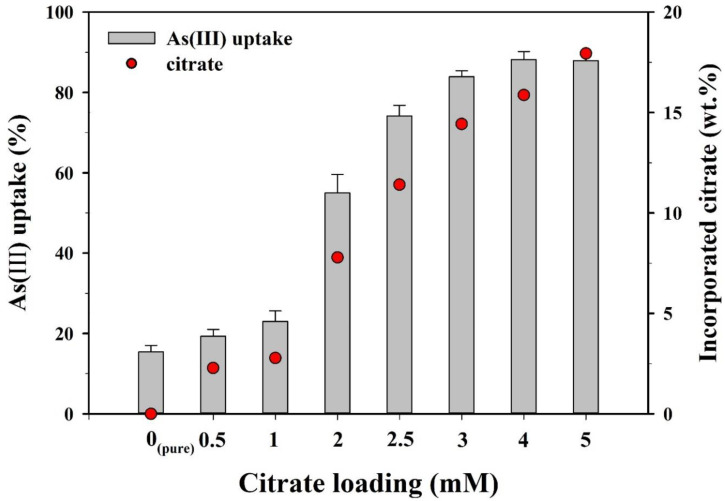
As(III) uptake by CF-NCs as a function of a loading rate of citrate.

Prior to the kinetics and isotherm experiments, batch sorption experiments were conducted to compare the As(III) sorption capacities of the CF-NCs synthesized with different loading rates of citrate (0–5 mM) (Figure 6). As the citrate loading increased from 0 to 1 mM, As(III) uptake increased slightly from 15.4% to 23.0%. As(III) uptake sharply increased from 55.0% to 83.9% over a range of 2–3 mM of citrate and reached a plateau (87.9–88.2%) at 4–5 mM. Sorption kinetics and isotherm experiments for As(III) and As(V) were examined using the CF-NCs synthesized with 5 mM of citrate, which showed the maximum sorption capacity.

#### 3.3.2. Sorption Kinetics for As(III) and As(V)

**Figure 7:**
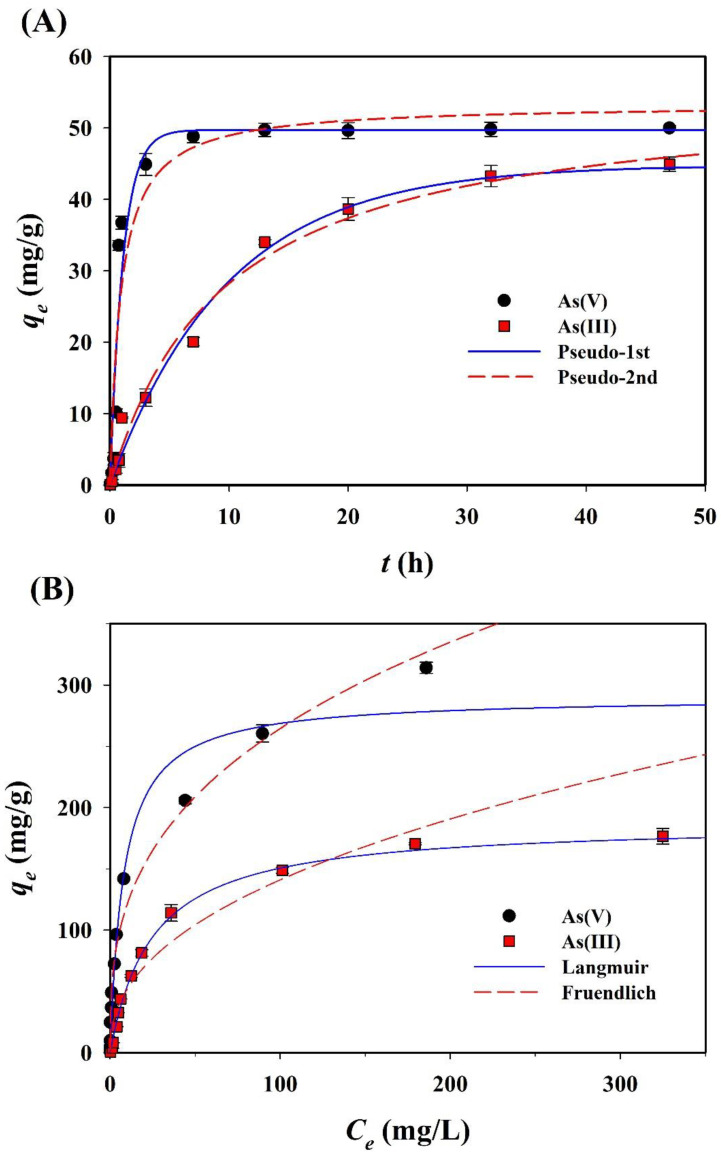
Experimental and plotted data of (**A**) sorption kinetics and (**B**) isotherms for As(III) and As(V) sorption on the CF-NCs.

The sorption of both arsenic species (As(III) and As(V)) onto CF-NCs was evaluated as a function of the contact time so as to confirm the kinetic sorption properties of both species and determine the optimal contact time for equilibrium (Figure 7A). As(III) sorption reached a plateau after 48 h of contact time (achieving over 97% of the maximum sorption capacity). Based on this result, a contact time of 48 h was chosen for the sorption isotherm experiments. The kinetics of As(III) and As(V) sorption were analyzed using pseudo-first-order and pseudo-second-order models. The non-linear fits to the data corresponding to the kinetic constants are summarized in Table 2. Compared to the pseudo-second-order counterpart, for As(III) sorption, the pseudo-first-order model resulted in a higher *R*^2^ (0.990) and a lower RMSE (1.906). The calculated adsorption capacity (*q_e_*_,cal_), 44.79 mg/g, obtained from the pseudo-first-order model agrees with the experimental adsorption capacity (*q_e_*_,exp_) of 44.90 mg/g. In the same manner, the pseudo-first-order model explains the As(V) sorption kinetics data better than the pseudo-second-order model.

#### 3.3.3. Sorption Isotherms for As(III) and As(V)

Arsenic sorption isotherms of CF-NCs were evaluated using the Langmuir and Freundlich models. The isotherm curves and parameters are presented in Figure 7B and Table 3, respectively. For As(III) sorption, the Langmuir model yielded better fits with the experimental data (*R*^2^ = 0.996, RMSE = 4.57) relative to the Freundlich model (*R*^2^ = 0.974, RMSE = 17.66). In contrast, for As(V) sorption, the Freundlich model yielded better fits with the experimental data (*R*^2^ = 0.981, RMSE = 15.44) than the Langmuir model (*R*^2^ = 0.969, RMSE = 19.55). These findings suggest that the As(III) sorption process on CF-NCs would follow a monolayer and homogeneous adsorption, whereas the As(V) sorption process would follow a multilayer and heterogeneous (ad)sorption. The theoretical maximum (ad)sorption capacities (*q_m_*), which were calculated using the Langmuir model under the experimental conditions for As(III) and As(V) sorption onto CF-NCs, were 188.97 and 290.22 mg/g, respectively. These values are considerably higher than the values reported in several previous studies (Table 4), which focused on the asenic (III and/or V) sorption by siderite-based sorbents under oxic and/or anoxic conditions [60,61].

## 4. Sorption Mechanisms of As(III) and As(V)

### 4.1. Solid- and Liquid-Phase Analyses

To elucidate the possible arsenic (III and V) sorption mechanism(s) onto CF-NCs, a series of solid- and liquid-phase analyses, including SEM/EDS, XRD, HPLC, ICP-OES, and XPS, were performed on the arsenic-sorbed samples.

**Figure 8:**
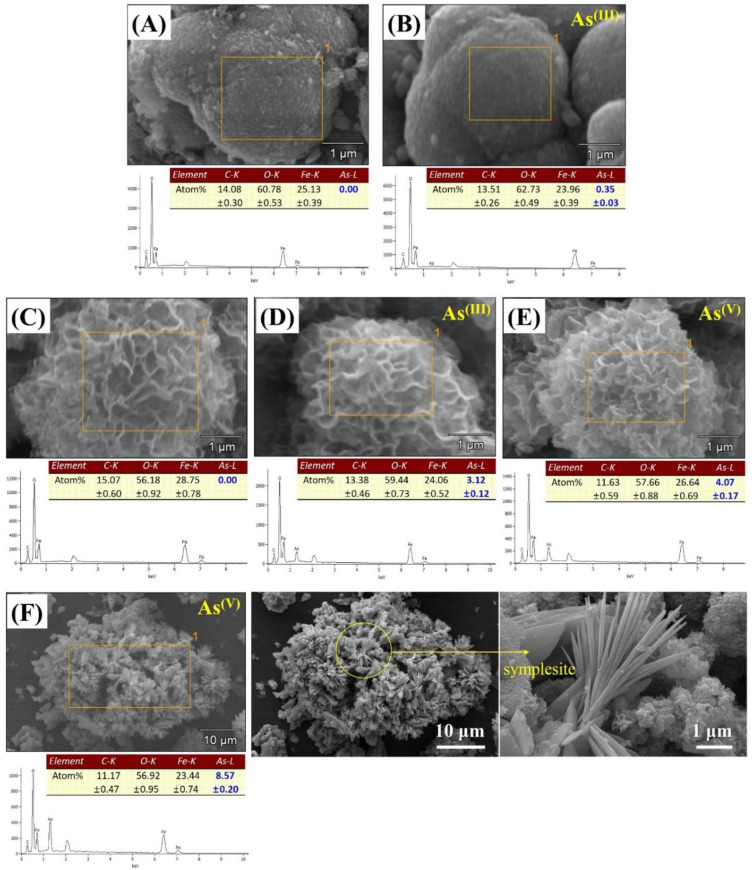
SEM/energy dispersive X-ray spectroscopy (EDS) results for the CF-NC samples before and after arsenic (III and V) sorption. (**A**) siderite, (**B**) As(III)-sorbed siderite, (**C**) CF-NCs, (**D**) As(III)-sorbed CF-NCs, and (**E**,**F**) As(V)-sorbed CF-NCs; (**F**) shows As(V)-sorbed CF-NCs with new precipitate (symplesite).

The SEM/EDS results of the arsenic-sorbed samples are presented in Figure 8. As shown in the EDS results, the atomic percent (at.%) of arsenic (*As-L*) at the siderite surface was slightly increased from 0 to 0.35 at.% after As(III) sorption (Figure 8A,B), whereas 3.12 at.% of arsenic (*As-L*) was detected at the surface of CF-NCs after As(III) sorption without any significant changes in their hierarchical 3D structure (Figure 8C,D). These observations are consistent with the enhanced As(III) uptake by CF-NCs in our batch sorption experiments. Similar to the As(III)-sorbed CF-NCs, a hierarchical surface structure with high As content (4.07 at.%) was observed in the As(V)-sorbed CF-NCs, which indicates that surface adsorption plays an important role in As(III) and As(V) removal by CF-NCs (Figure 8E). Additionally, some flat and lath-like crystals containing large amounts of As (8.57 at.%) were newly observed after As(V) sorption (yellow circle in Figure 8F). With XRD analysis (Figure S3), additional precipitates were not observed in the As(III)-sorbed samples, whereas a new XRD pattern was observed in the As(V)-sorbed samples. A new phase was confirmed as a ferrous arsenate mineral, symplesite (Fe^2+^_3_(As^(V)^O_4_)_2_·8H_2_O). Similar to the results presented herein, it has been reported that symplesite is formed using structural Fe(II)-carbonate as a sorbent during As(V) sorption under anoxic conditions [60].

**Figure 9:**
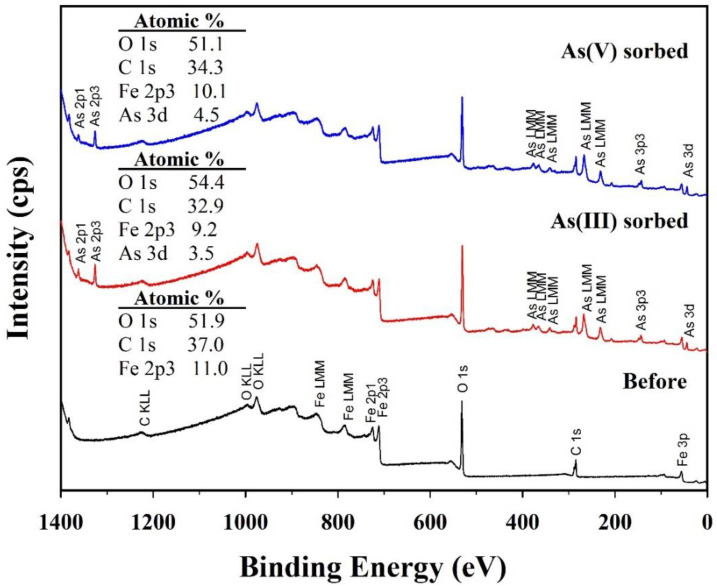
X-ray photoelectron spectroscopy (XPS) survey spectra for the CF-NC samples before and after arsenic (III and V) sorption.

Chemical compositions of solid and solution samples before and after arsenic (III and V) sorption were further analyzed to clarify the formation mechanism of symplesite using XPS, HPLC, and ICP-OES. Significant changes in the chemical compositions of the solid samples were confirmed in the XPS survey spectra (Figure 9). The CF-NCs before arsenic sorption had Fe of 11.0 at.%, C of 37.0 at.%, and O of 51.9 at.%. After arsenic (III and V) sorption, As was detected, with values of 3.5 at.% and 4.5 at.%, respectively. These results agree well with the EDS results for the As(III)- and As(V)-sorbed samples, indicating successful arsenic sorption on CF-NCs. However, the Fe content in the CF-NCs decreased to 9.2 at.% and 10.1 at.% after As(III) and As(V) sorption, respectively. In addition, HPLC and ICP-OES showed the presence of citrate and Fe in the residual solutions reacted with CF-NCs (Table 5). These XPS, HPLC, and ICP results indicate the dissolution of CF-NCs during the (ad)sorption reaction, which is attributed to the amorphous feature of CF-NCs caused by the incorporation of the soluble (Fe^2+^citrate)^−^ complexes [59,60]. Interestingly, despite the similar concentration of citrate between the two residual solutions of As(III) and As(V), the concentration of Fe in the residual solution after As(V) sorption is significantly lower by 23.5 mg/L than that of the solution after As(III) sorption. This difference can be explained by the precipitation of the symplesite, as confirmed by SEM and XRD analyses earlier during As(V) sorption.

### 4.2. X-ray Absorption Spectroscopy (XAS) Analysis

**Figure 10:**
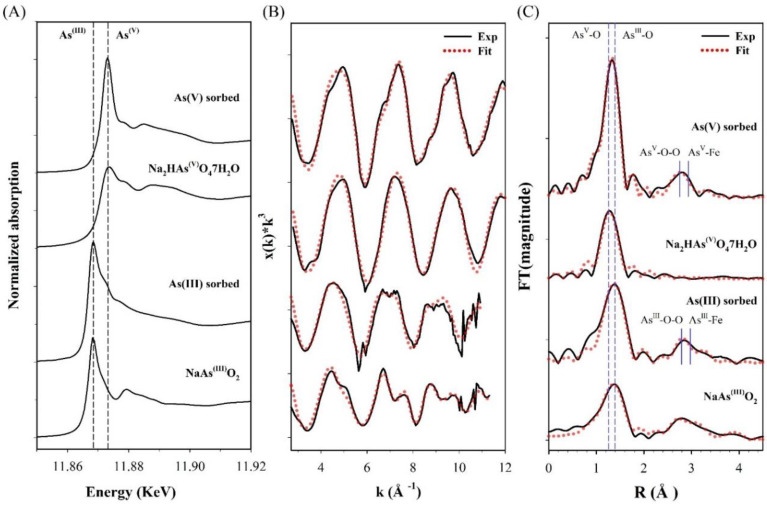
X-ray absorption spectroscopy (XAS) analysis for the arsenic (III and V)-sorbed CF-NCs and reference samples. (**A**) Normalized X-ray absorption near edge structure (XANES) spectra, (**B**) k^3^-weighted χ(k) extended X-ray absorption fine structure (EXAFS) spectra, and (**C**) Fourier transform of the χ(k) EXAFS spectra.

To further elucidate the arsenic (III and V) sorption mechanisms on CF-NCs, XAS analysis was performed on the arsenic (III and V)-sorbed CF-NCs and reference samples, and the results are shown in Figure 10. The position of the sharp intense absorption peaks “white line” (dashed line) for the arsenic-sorbed samples in the normalized arsenic K-edge XANES showed no changes with respect to the peaks of As(III) reference sample, NaAsO_2_ (11.868 KeV), and As(V) reference sample, Na_2_HAsO_4_·7H_2_O (11.874 KeV) (Figure 10A), indicating no oxidation changes in the sorbed arsenic species. These observations are consistent with previous studies reporting no evidence of oxidation of sorbed arsenic on siderite, green rust, and a Fe(II)-carbonate sorbent under anoxic conditions [23,28,60]. Therefore, we note that both As(III) and As(V) are sorbed on CF-NCs without any oxidation changes.

The coordination environments of As(III) and As(V) bound on CF-NCs were further characterized using EXFAS measurements. The χ(k^3^) EXAFS spectra and their Fourier transformed data in R-space for the arsenic (III and V)-sorbed CF-NCs and reference samples are presented in Figure 10B,C, respectively. The shell fitting results for the EXAFS data are listed in Table 6. As seen in Figure 10C, the first predominant shell of the As(III)-sorbed CF-NCs and of an NaAsO_2_ are due to the As-O scattering and are fitted with three oxygen atoms (CN = 3, based on the XANES results), where the distance of the As-O scattering (R) is 1.78 Å. These values are well consistent with the known value of As(III) sorbed on siderite with CN = 2.8–3.0 and R = 1.78 Å under unoxic conditions, indicating that three oxygen atoms were coordinated around an As atom at a distance of 1.78 Å (pyramidal configuration of AsO_3_) [23,24]. The second shell of the EXAFS data of the As(III)-sorbed CF-NCs was attributed to the As−O−O multiple scattering and the As−Fe scattering. The As–Fe scattering of the As(III)-sorbed CF-NCs was fitted with 1.22 Fe atom (CN = 1.22) at R = 3.39 Å, which is close to the As–Fe distance of 3.35 Å with 1.8 Fe atom corresponding to bidentate binuclear corner-sharing complexes of As(III) on siderite under anoxic conditions [24]. Compared to the CN of 2 at the As–Fe shell (assuming bidentate binuclear inner-sphere complexes only), however, the lower CN for the As(III)-sorbed CF-NCs (=1.22) indicates that As(III) adsorption on CF-NCs via outer-sphere complexation could partially contribute to the As(III) removal from the solution. This is in good agreement with a previous report stating that As(III) is adsorbed on siderite through outer-sphere complexation [23].

**Figure 11:**
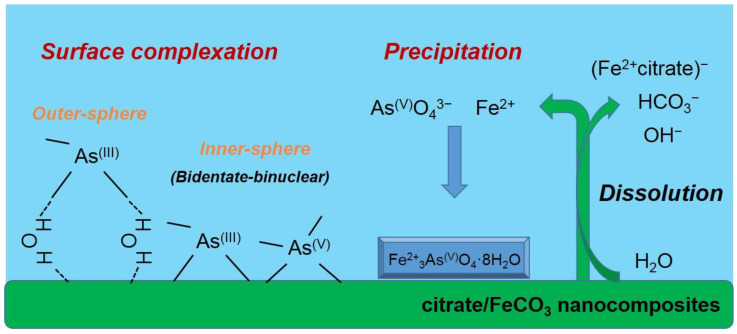
Schematic illustration of suggested mechanism(s) of arsenic (III and V) uptake by CF-NCs.

As(V)-sorbed CF-NCs and an As(V) reference sample (Na_2_HAsO_4_·7H_2_O) could be fitted with four oxygens atoms (CN = 4) at 1.70 and 1.69 Å As-O distance, respectively. These values are consistent with those of As(V) adsorbed on siderite with CN = 4 and R = 1.69 Å, indicating that four oxygen atoms were coordinated around an As atom at a distance of 1.70 Å (tetrahedron configuration of AsO_4_). The As–Fe scattering of the As(V)-sorbed CF-NCs was fitted with 3.91 Fe atom at R = 3.37 Å, where the distance R is very close to the reported values R = 3.34–3.36 Å with CN = 2.0 under unoxic conditions [23,24], indicative of bidentate-binuclear corner-sharing inner-sphere complexes of the AsO_4_ tetrahedra. However, the higher CN value of 3.91 for the As(V)-sorbed CF-NCs relative to CN = 2 (assuming bidentate binuclear mode only) shows the existence of other As–Fe bonding forms. These explanations are better elucidated by the precipitation of symplesite (Fe_3_(AsO_4_)_2_·8H_2_O), which was observed after As(V) sorption under anoxic conditions [23,28]. The reported distance of the second shell (As–Fe) of the symplesite ranges widely from 3.31 to 3.55 Å with CN = 5 [23]. The presence of symplesite can result in slightly longer R and larger CN values (R = 3.37 Å with CN = 3.91) than adsorption alone. These XRD, SEM/EDS, and EXAFS results suggest that in addition to inner-sphere surface complexation, precipitation of symplesite cannot be ruled out as mechanism(s) of As(V) uptake by CF-NCs. A schematic illustration of the investigated arsenic (III, V) removal mechanism(s) using CF-NCs under anoxic conditions is presented in Figure 11.

## 5. Conclusions

In this study, the synthesis of hierarchically structured citrate/FeCO_3_ nanocomposites (CF-NCs) was successfully demonstrated, and the mechanism(s) of citrate incorporation into siderite as a function of citrate concentration were investigated systematically. The experiments and investigations presented herein are the first in which arsenic (III and V) uptake by organic-incorporated siderite in anoxic condition was characterized. As(III) sorption is highly dependent on the content of incorporated citrate in CF-NCs. Therefore, it is worth noting that As(III) can be effectively attenuated by siderite formed in the presence of organic compounds such as citrate, even under anoxic subsurface environments. Both arsenic species, As(III) and As(V), can be sorbed onto CF-NCs predominantly via a bidentate corner-sharing surface complexation, which can accompany a limited outer-sphere surface complexation for As(III), including precipitation of symplesite for As(V). These results provide valuable information on the use of siderite-based materials as sorbents and can help to establish an effective method for As(III) attenuation in subsurface environments.

## Figures and Tables

**Table 1:** Chemical composition of the CF-NC samples synthesized in the presence of different citric acid concentrations (0–5 mM).

^a^ [Citrate]_ini_ in Solution (mM)	Chemical Composition (wt.%)		
	^b^ Citrate	^c^ Fe	^c^ CO_3_
0	-	48.21	51.79
0.5	2.28	47.63	50.09
1	2.78	47.51	49.71
2	7.78	46.24	45.98
2.5	11.41	45.33	43.26
3	14.43	44.56	41.01
4	15.87	44.20	39.93
5	17.94	43.68	38.38

^a^ Initial concentration of citrate in the synthetic solution. ^b^ Amount of citrate in the synthesized CF-NC samples measured by HPLC after acidification of the known mass of CF-NC sample using 1 M HCl. ^c^ Amount of Fe and CO_3_ in the synthesized CF-NC samples calculated by using the formula: xFeCO_3_ + yFe_3_(citrate^3−^)_2_ = 100, x + y = 1.

**Table 2:** Sorption kinetics parameters for As(III) and As(V) using CF-NCs as sorbents.

Models	Parameters	As(III)	As(V)
Experimental	*q_e,_*_exp_ (mg/g)	44.90	49.98
Pseudo-1st-order	*q_e,_*_cal_ (mg/g)	44.79	49.72
	*k*_1_ (1/h)	0.101	0.922
	*R* ^2^	0.990	0.937
	RMSE	1.906	5.577
Pseudo-2nd-order	*q_e,_*_cal_ (mg/g)	55.26	53.31
	*k*_2_ (g/mg h)	0.00189	0.0210
	*R* ^2^	0.989	0.914
	RMSE	1.937	6.540

**Table 3:** Sorption isotherm parameters for As(III) and As(V) using CF-NCs as sorbents.

Models	Parameters	As(III)	As(V)
Langmuir	*q_m_* (mg/g)	188.97	290.22
	*k_L_* (L/mg)	0.0404	0.1243
	*R* ^2^	0.996	0.969
	RMSE	4.57	19.55
Freundlich	*k*_F_ (mg/g (L/mg)^1/n^)	22.64	55.25
	1/n	0.3770	0.3401
	*R* ^2^	0.974	0.981
	RMSE	17.66	15.44

**Table 4:** Comparison of sorption capacities for arsenic (III and V) among different siderite-based sorbents.

Experimental Conditions (at Room Temperature, Neutral pH)						Maximum Sorption Capacity (*q_m_*) * (mg/g)	References
Arsenic Species		Siderite Types (M = Modified)		Oxygen Conditions			
As (III)	As (V)	Natural (N)	Synthetic (S)	Oxic (O)	Anoxic (A)		
III			MS		A	188.97	This study
	V		MS		A	290.22	
III		N		O		0.52	Guo et al. (2007) [22]
	V	N		O		1.04	
	V	MN		O		2.19	Zhao and Guo (2014) [49]
III			S		A	10.2	Guo et al. (2013) [24]
III			S	O		115.0	
	V		S		A	10.7	
	V		S	O		121.0	
III		MN		O		9.43	Zhao et al. (2014) [25]
III			S	O		58.7	Hahhi et al. (2019) [29]
	V		S	O		10.7	Guo et al. (2010) [62]
III		MN		O		8.22	Li et al. (2017) [63]
III			S	O		9.98	Guo et al. (2011) [27]

Note: Theoretical maximum sorption capacity (*q_m_*)* calculated by Langmuir model.

**Table 5:** Solution chemistry after reaction with CF-NCs.

Solution Type	[Citrate] in Solution (mM)	[Fe] in Solution (mM)	ORP (mV)
Control	0.777 (=149.3 mg/L)	1.270 (=70.9 mg/L)	−356
As(III) solution	0.896 (=172.1 mg/L)	1.430 (=79.9 mg/L)	-
As(V) solution	0.903 (=173.5 mg/L)	1.010 (=56.4 mg/L)	-

Note: Control sample was reacted only with DO-free water without arsenic. Citrate and Fe concentrations were measured by HPLC and ICP-OES, respectively. ORP (oxidation reduction potential) was measured by pH meter equipped with an ORP electrode.

**Table 6:** Shell fitting results for the arsenic (III and V)-sorbed CF-NCs and reference samples.

Sample	Path	^a^ CN	^b^ R(Å)	^c^ σ^2^(Å^2^)	^d^*E_0_*(eV)	^e^ *R_f_*
As(V) sorbed	As-O	4.09	1.70	0.0025	8.2	0.16
	As-O-O	7.10	3.16	0.0052		
	As-Fe	3.91	3.37	0.0138		
Na_2_HAsO_4_∙7H_2_O	As-O	4 *	1.69	0.0019	9.7	0.10
	As-O-O	15.2	2.93	0.0027		
As(III) sorbed	As-O	3 *	1.78	0.0041	11.0	0.26
	As-O-O	9.98	3.19	0.0015		
	As-Fe	1.22	3.39	0.0112		
NaAsO_2_	As-O	3 *	1.78	0.0094	9.5	0.20
	As-As	3.10	3.26	0.0108		
	As-Na	2.34	3.34	0.0216		
	As-As	11.37	4.09	0.0282		

Note: ^a^ CN = Coordination number, ^b^ R = Interatomic distance, ^c^ σ^2^ = Debye-Waller factor, ^d^ *E*_0_ = Energy shift, ^e^ *R_f_* = *R*-factor, * Fixed value. The estimated errors for the 1st shell are ± 25% for CN, ± 0.03 Å for R and ± 0.002 Å^2^ for σ^2^, and for 2nd and 3rd shell ± 60% for CN, ± 0.1 Å for R, and ± 0.01 Å^2^ for σ^2^.

## Supplementary Materials

The following are available online at https://www.mdpi.com/2079-4991/10/9/1773/s1, Figure S1. Schematic illustration for the synthesis procedure of citrate/FeCO_3_ nanocomposites, Figure S2. Modelling data of (A) the distribution of citrate species without iron and carbonate ions as a function of pH, (B) the distribution of citrate species as a function of citrate concentration, (C) the distribution of Fe species, and (D) the saturation index (SI) of siderite at different citrate concentrations calculated by PHREEQC software with the minteq.v4 database, Figure S3. XRD patterns for the CF-NCs samples before and after arsenic (III and V) sorption.
