# Supplementary material for: Enhanced Arsenic (III and V) Removal in Anoxic Environments by Hierarchically Structured Citrate/FeCO3 Nanocomposites
Source: Nanomaterials (Basel). 2020 Sep 8;10(9):1773. doi: 10.3390/nano10091773 (PMC7558564; doi:10.3390/nano10091773)
Supplement: Supplementary file 1 [file nanomaterials-10-01773-s001.pdf]

Supplementary Materials

Enhanced Arsenic (III and V) Removal in Anoxic Environments by Hierarchically Structured Citrate/$FeCO_3$ Nanocomposites

Seon Yong Lee [1], YoungJae Kim [2], Bongsu Chang [1], and Young Jae Lee [1,*]

[1] Department of Earth and Environmental Sciences, Korea University, 145 Anam-ro, Seongbuk-gu, Seoul 02841, Republic of Korea; reddevels86@korea.ac.kr; bschang@korea.ac.kr; youngjlee@korea.ac.kr
[2] Chemical Sciences and Engineering Division, Argonne National Laboratory, 9700 South Cass Avenue, Lemont, Illinois 60439, United States; youngjkm@anl.gov
* Correspondence: youngjlee@korea.ac.kr; Tel.: +82-2-3290-3181, Fax: +82-2-3290-3189

Pages: 4

Figures: 3

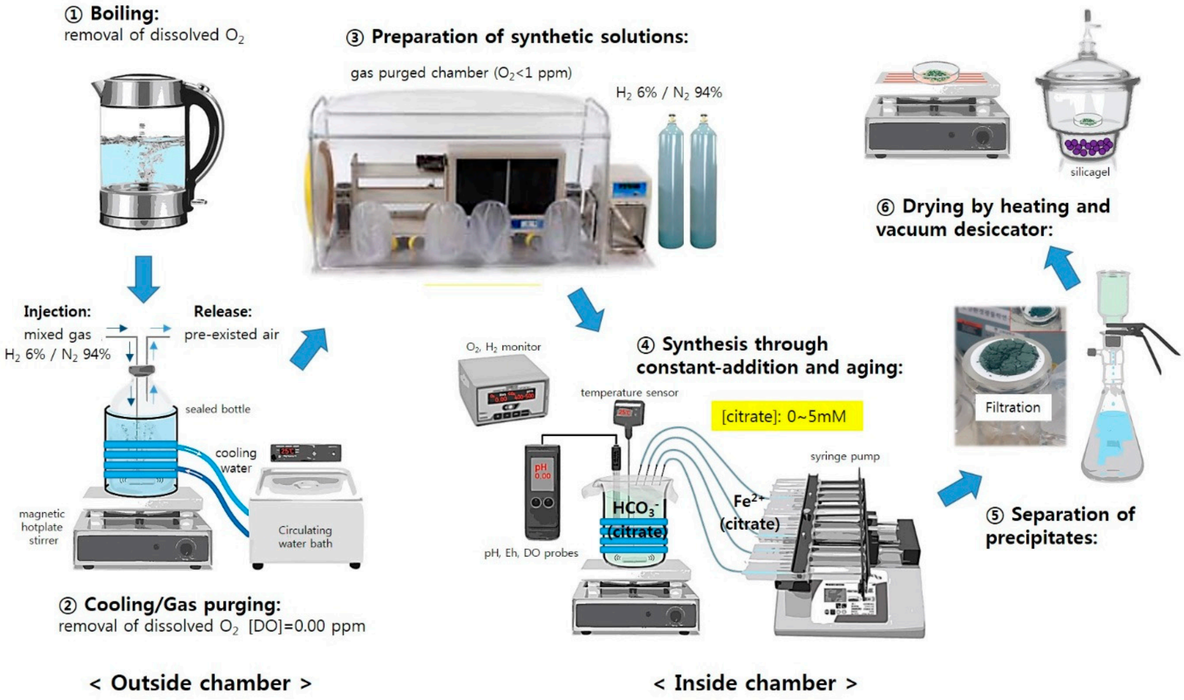

Figure S1. Schematic illustration for the synthesis procedure of citrate/$FeCO_3$ nanocomposites.

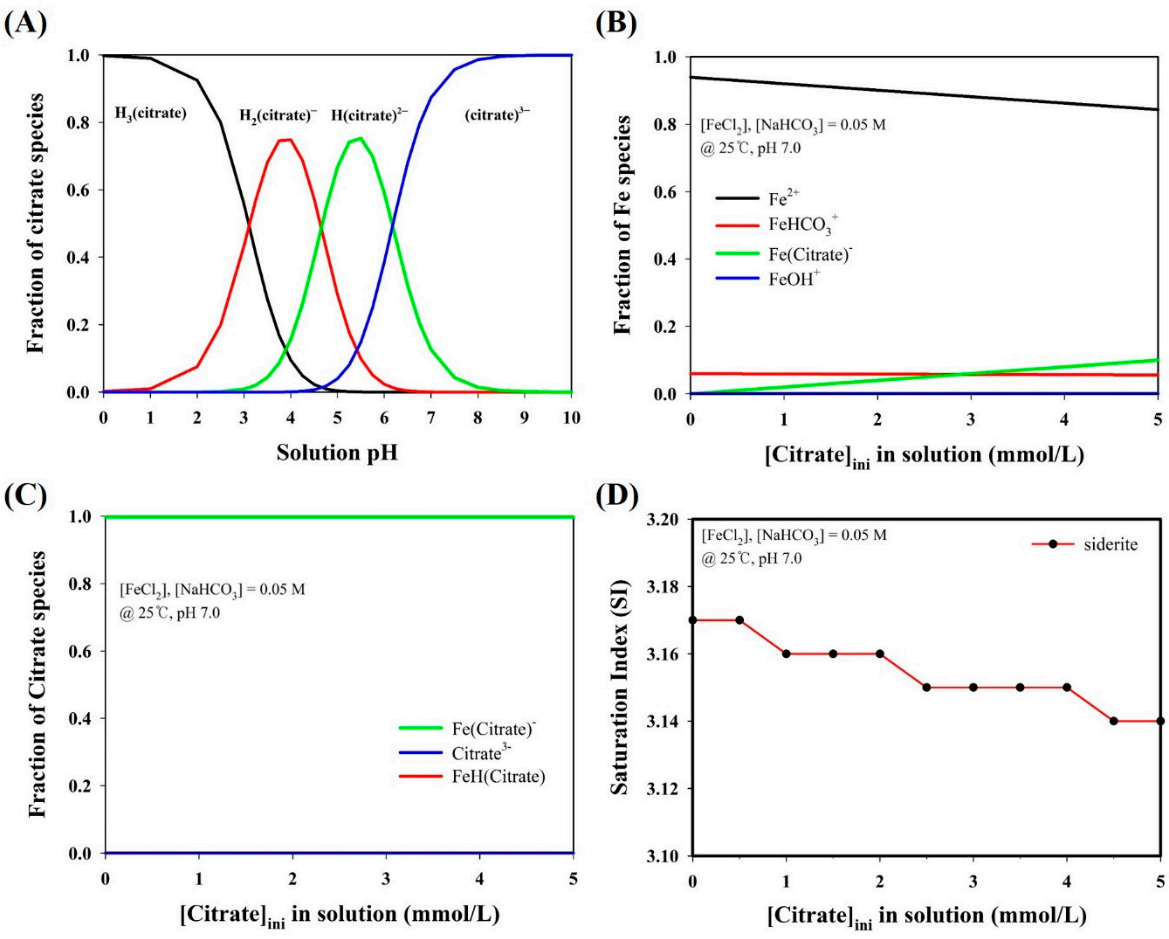

Figure S2. Modelling data of (A) the distribution of citrate species without iron and carbonate ions as a function of pH, (B) the distribution of citrate species as a function of citrate concentration, (C) the distribution of Fe species, and (D) the saturation index (SI) of siderite at different citrate concentrations calculated by PHREEQC software with the minteq.v4 database.

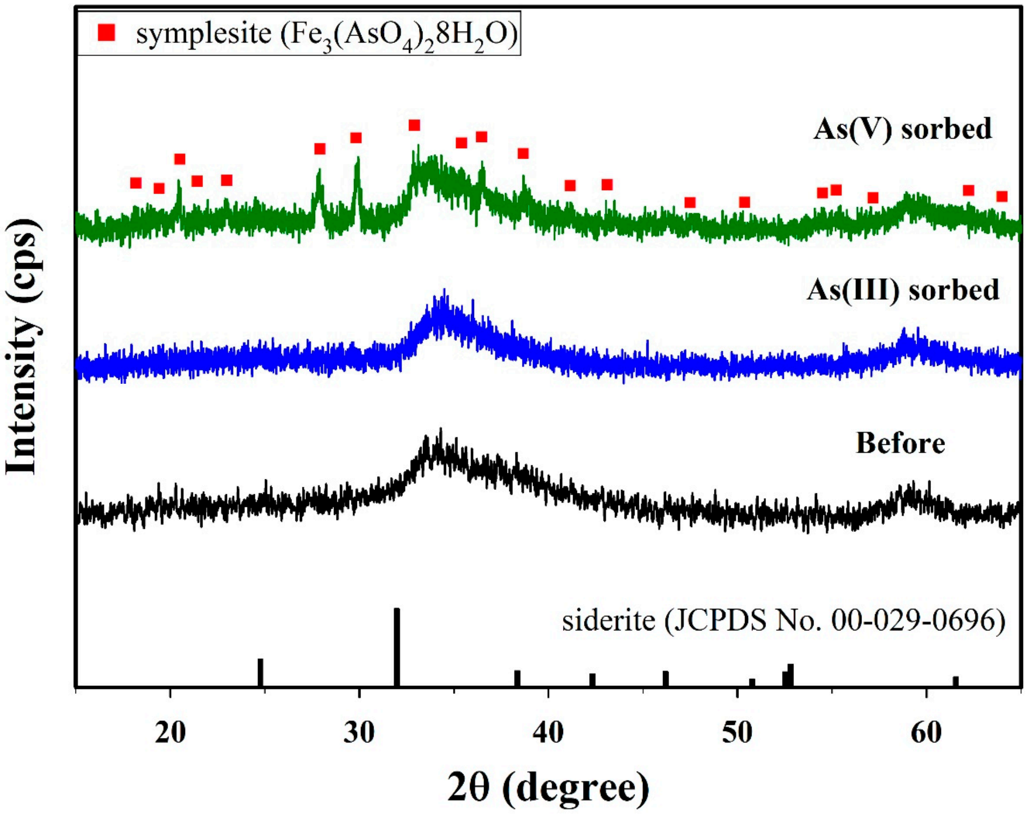

Figure S3. XRD patterns for the CF-NCs samples before and after arsenic (III and V) sorption.
